# A minimally invasive tubular retractor–assisted retropleural approach for thoracic disc herniations — case series and technical note

**DOI:** 10.1007/s00701-022-05470-w

**Published:** 2023-01-18

**Authors:** Vanessa Hubertus, Peter Selhausen, Franziska Meinert, Frerk Meyer, Julia S. Onken, Ulf C. Schneider, Nils Hecht, Marcus Czabanka, Peter Vajkoczy, Johannes Woitzik

**Affiliations:** 1 Department of Neurosurgery, Charité–Universitätsmedizin Berlin, corporate member of Freie Universität Berlin, Humboldt-Universität Zu Berlin, Berlin Institute of Health, Berlin, Germany; 2 Department of Neurosurgery, Universitätsklinikum Hamburg-Eppendorf, Hamburg, Germany; 3 Department of Neurosurgery, Carl Von Ossietzky Universität Oldenburg, Oldenburg, Germany; 4 Department of Spine Surgery, Carl Von Ossietzky University Oldenburg, Oldenburg, Germany; 5 Department of Neurosurgery, Luzerner Kantonsspinal, Lucerne, Switzerland; 6 Department of Neurosurgery, Goethe Universität Frankfurt/Main, Frankfurt/Main, Germany; 7 Research Center Neurosensory Science, Carl Von Ossietzky Universität Oldenburg, Oldenburg, Germany; 8 Department of Neurosurgery, Evangelisches Krankenhaus Oldenburg, Marienstr. 11, Oldenburg, 26121 Germany

**Keywords:** Thoracic disc herniation, Minimally invasive spine surgery, Thoracic spine surgery, Tubular retractor–assisted surgical approach, Retropleural dissection

## Abstract

**Purpose:**

Thoracic disc herniations are uncommon and carry a high risk for neurological deterioration. Traditional surgical approaches include thoracotomy, costotransversectomy or posterior approaches with considerable morbidity. In this technical note with case series, we describe a minimally invasive tubular retractor–assisted retropleural approach for simple and less invasive microsurgical exploration of thoracic disc herniations from a lateral angle.

**Methods:**

Surgical technique consisted of partial rib resection and retropleural dissection followed by the placement of a tubular retractor (METRx Tubes, Medtronic) for an anterior-lateral exposure of the disc and neuroforamen. Epidemiological, clinical and surgical patient data were acquired.

**Results:**

Between 2017 and 2020, six patients were surgically treated using the minimally invasive tubular retractor–assisted retropleural approach. Microsurgical exposure of the disc and neural structures was achieved from a lateral direction without requiring thoracotomy or lung deflation. Control imaging confirmed resection in all cases without relevant residuum. As postoperative complications, one dural injury and one postoperative pneumothorax occured. No neurologic deterioration or recurrence occurred during a median follow-up of 3 months.

**Conclusion:**

The described tubular retractor–assisted retropleural exposure serves as a feasible minimally invasive microsurgical approach to the anterior-lateral thoracic spine.

## Introduction

Symptomatic disc herniations in the thoracic spine are uncommon and occur only in up to 3% of all patients suffering from disc herniations [8, 9]. Mainly, they are located in the mid- to lower thoracic spine and are prone to neurological deficits in up to 60% [6, 8, 19, 23]. To surgically treat thoracic disc herniations, anterior, posterior, and lateral approaches are available. Common anterior approaches include transthoracic, transsternal and thoracoscopic approaches, whilst common posterior approaches are transpedicular and trans-facet pedicle sparing approaches, and traditional lateral approaches include lateral extracavitary approaches and costotransversectomy [2, 4, 8, 24]. Lateral retropleural approaches to the thoracic spine give the advantage of using the shortest way to the anterior-lateral thoracic spine without opening the pleural cavity [1, 13, 16, 24]. In the past years, case series described refinements of this lateral approach to the thoracic spine for the treatment of thoracic disc herniations, combining the technique with neuronavigation, videoendoscopy, or retractor-assisted approaches using an X-LIF retractor system, allowing for an enhanced exposure of thoracic disc herniations whilst decreasing surgical morbidity [1, 8, 13, 17, 22, 24]. With this report, we describe a minimally invasive tubular retractor–assisted retropleural approach which allows for a microsurgical exploration and resection of thoracic disc herniations from a lateral angle in a series of six patients.

## Materials and methods

### Clinical and surgical data

Patients with symptomatic one-level thoracic disc herniations surgically treated between 2017 and 2020 using a new minimally invasive tubular retractor–assisted approach were included in this case series. Patient recruitment and surgery were performed at two academic institutions. Included patients suffered from symptomatic one-level disc herniations at the mid– to lower thoracic level (Th6/7—Th10/11) which were lateralized and at least partially calcified. Clinical, surgical and imaging data was analyzed restropectively. Patients received postoperative routine treatment including early mobilisation and physiotherapy. Postoperative control imaging was performed to assess disc herniation removal using MRI or CT. All patients received routine clinical follow-up.

*Ethical statement.*

This case series was conducted according to the World Medical Association Declaration of Helsinki. The study was approved by the local ethics committee and informed patient consent was waived due to the retrospective nature of the study (Ethics committee approval by the Charité-Universitätsmedizin Berlin, Germany, number EA4/064/20).

### Data management

Data management was performed using Microsoft Excel and internal file servers. Descriptive statistical analysis was performed using GraphPad Prism 8 (GraphPad Software, La Jolla, CA, USA).

## Results

### Technical note to the surgical technique

**Fig. 1:**
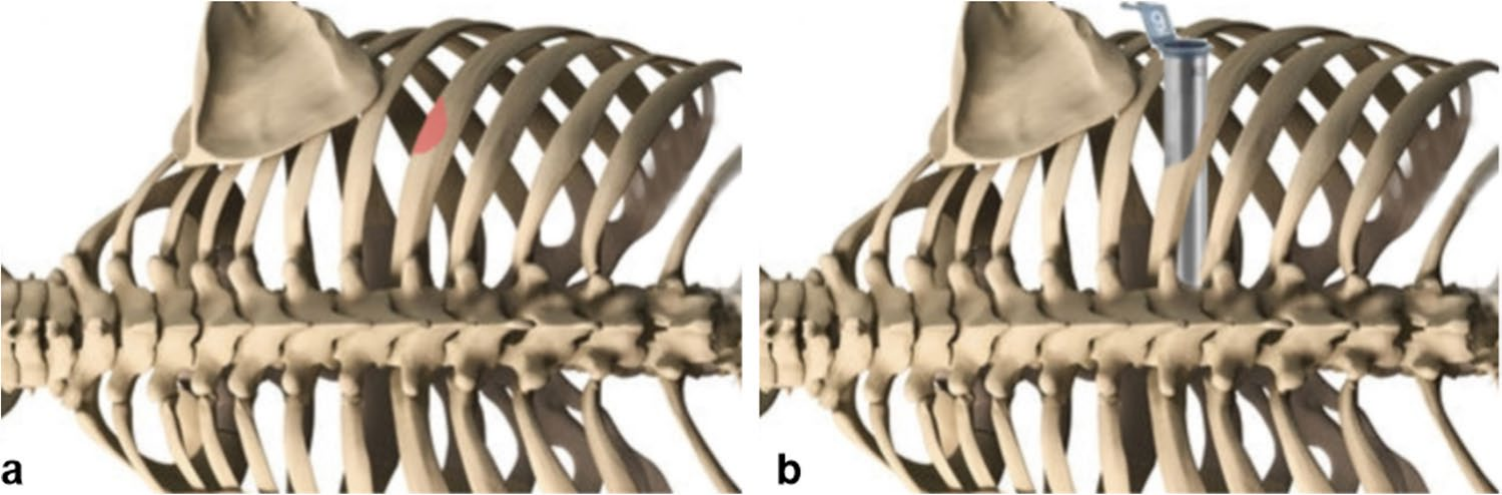
Schematic illustration of partial resection of the lower rib (**a**) and positioning of the tubular retractor (**b**) for the retropleural, retractor-assisted approach to thoracic disc herniations

**Fig. 2:**
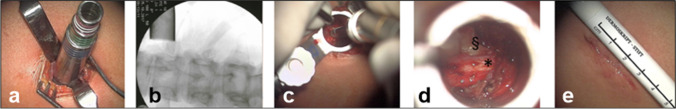
Insertion of the tubular retractor (**a**) with the aid of intraoperative fluoroscopy at the level Th 10/11 (**b**) and drilling of the head of the rib and posterior lateral part of the disc (**c**) allows removal of the disc herniation with optimal anterior/lateral exposure of the disc space (**d**,§) and dura (**d**,*). (**e**) illustrates the wound after closure

Surgery was performed under general anesthesia. A double lumen tube for respiration was inserted to allow for unilateral lung deflation during retropleural dissection. Antibiotic prophylaxis via single-shot infusion of Cefazolin (2 g) was applied 30 min prior to the skin incision. The patient was positioned laterally on an X-ray transparent operating table (Trumpf Carbon Spine tabletop, Trumpf Medical), using a deflatable mattress. The side of the herniated disc was facing up. The arm was elevated and positioned at 90° from the thorax. Fluoroscopic localization of the index level was performed anterior-posteriorly and laterally and the thus localized index level was marked on the skin using a water-proof marker. A 3 cm skin incision was applied at about 12 cm paramedian on the side of the herniated disc. Following retractor-insertion under fluoroscopic control, the caudal rib of the index segment was partially resected using a diamond drill (Fig. 1a). Retropleural dissection under short apnea and unilateral lung deflatation was performed to expose the head of the caudal rib and the neuroforamen of the index segment. Next, a tubular retractor (METRx Tubes, Medtronic) was placed through this retropleural corridor using fluoroscopic control (Fig. 1b). The tubular retractor was placed over the disc and the neuroforamen (Fig. 1b, Fig. 2a, b). This approach allows for the microsurgical exploration and removal of the disc herniation whilst providing an excellent view of the disc and the neural structures from an anterior-lateral view (Fig. 2c, d). Following the removal of the disc herniation and meticulous hemostasis, the tubular retractor was removed without the necessity for another apnoe episode and the skin was closed in a layering fashion, with dermabond skin glue applied for external closure (Fig. 2e) (surgical steps: Fig. 2, Table 1).

**Table 1:** The nine surgical steps of the minimally invasive tubular retractor–assisted retropleural approach for the thorough exploration and removal of thoracic disc herniations from an anterior-lateral direction

Surgical step	Description
1. Patient positioning	The patient is positioned laterally, with the affected side facing up
2. Index localization	Localization of the index level is performed using fluoroscopic control
3. Skin incision	A skin incision measuring approximately 3 cm is applied at approximately 12 cm paramedian
4. Partial rib resection	The caudal rib of the index segment is resected using a diamond drill. (Fig. 1a) Beforehand, fluoroscopic control confirms the right level
5. Retropleural dissection	Retropleural dissection and exposure of the ipsilateral head of the caudal rib and the neuroforamen is performed
6. Tubular retractor insertion	The tubular retractor is inserted and positioned through the site of the partially resected lower rib using fluoroscopic control and is placed over the disc and neuroforamen during a short apnea and deflation of the ipsilateral lung. (Fig. 1b, Fig. 2a,b)
7. Exposure	Excellent exposure of the disc and neural structures is achieved from an anterior-lateral direction. (Fig. 2)
8. Exploration and Removal	The disc herniation can be thoroughly explored and removed from anterior-laterally
9. Closure	Following thorough hemostasis, wound closure is performed in a layering fashion with the skin closed using Dermabond® skin glue

### Case series

**Fig. 3:**
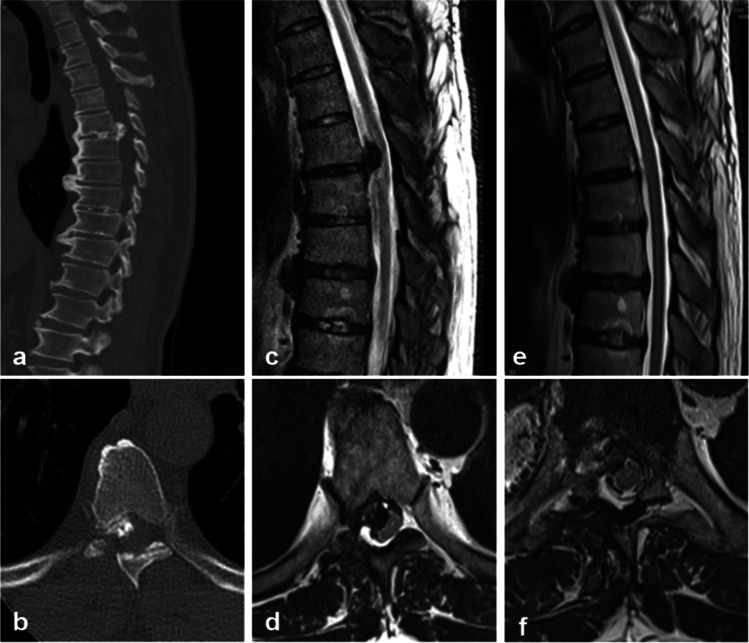
Preoperative (**a**–**d**) and postoperative (**e**–**f**) MRI of patient No. 5 with a mediolateral left-sided, partially calcified disc herniation at level Th 7/8 causing relevant spinal cord compression, with spinal cord and radicular compression completely removed in (**e**) and (**f**)

Between 2017 and 2020, six patients were surgically treated with symptomatic one-level thoracic disc herniations using the minimally invasive tubular retractor–assisted retropleural approach. All patients suffered from a symptomatic uni-level thoracic disc herniation at the mid- to lower thoracic level (Th6/7—Th10/11) which was lateralized and at least partially calcified. One patient suffered from a recurrent thoracic disc herniation and was previously surgically treated via posterior laminotomy. All patients suffered from lateralized radicular pain and 4/6 patients from neurological deficits. In most patients, symptoms persisted for more than 1 month (detailed data on epidemiology and clinical presentation: Table 2). The median duration of surgery was 159 (58–211) minutes. Perioperative blood loss was minimal and transfusion was not necessary (surgical data: Table 3). One patient suffered from an intraoperative dural injury which was fixed intraoperatively with primary dural suture, but was associated with the necessity of a lumbar drainage for four consecutive days to prevent dural fistula. Another patient suffered a postoperative pneumothorax with the necessity of a temporary chest tube which could be removed without complications within 3 days following surgery. Other common postoperative complications like surgical site infections or hematomas did not occur. Two patients complained of slight postoperative thoracalgia and skin irritation on the side of the approach, decreasing over the first postoperative days. Neurological deterioration did not occur and postoperative pain was improved. Postoperative MRI or CT was routinely performed to confirm the near total removal of the thoracic disc herniation, defined as no residual spinal canal stenosis or nerve root compression in postoperative imaging (example: Fig. 3). No neurological deterioration and no recurrent disc herniation occurred at a median follow-up of 3 months (detailed data on postoperative course and follow-up: Table 3).

**Table 2:** Epidemiological and clinical data of the enrolled patients

Patient No	Age (years)	Sex	BMI (kg/m^2^)	Level of disc herniation	Calcified	Lateralized	Previous surgery	Radicular pain	Neurological deficits	Acuteness of symptoms	Preoperative neurological scoring
1	40	F	24	Th 10/11	Yes	Left	No	Yes	None	Non-acute	AIS E, mJOA 18
2	55	F	25	Th 7/8	Yes	Left	No	Yes	None	Non-acute	AIS E, mJOA 18
3	52	M	33	Th 6/7	Yes	Left	Yes	Yes	Ataxia	Non-acute	AIS D, mJOA 15
4	60	F	23	Th 9/10	Yes	Left	No	Yes	Ataxia	Non-acute	AIS D, mJOA 15
5	31	M	23	Th 7/8	Yes	Left	No	Yes	Hypesthesia	Acute	AIS E, mJOA 17
6	54	F	36	Th 10/11	Yes	Right	No	Yes	Dysesthesia	Non-acute	AIS E, mJOA 18

*AIS*, ASIA (American Spinal Injury Association) Impairment Scale; *BMI*, body mass index; *F*, female; *M*, male; mJOA, modified Japanese Orthopedic Association score; *Th*, thoracic vertebra

**Table 3:** Surgical and outcome data of the enrolled patients

Patient No	Duration of surgery (min)	Blood transfusion	Perioperative complications	Chest tube (duration)	Clinical deterioration	Postoperative MRI/CT	Hospital stay (days)	Postoperative pain	Postoperative neurological scoring	Follow-up (months)
1	91	No	None	No	No	No residuum	4	Improved	AIS E, mJOA 18	2
2	180	No	None	No	No	No residuum	6	Improved	AIS E, mJOA 18	2
3	162	No	Pneumothorax	Yes (3 days)	No	No residuum	16	Improved	AIS E, mJOA 18	28
4	58	No	None	No	No	No residuum	6	No pain	AIS D, mJOA 15	12
5	155	No	None	No	No	No residuum	5	No pain	AIS E, mJOA 17	3
6	211	No	Dural injury	No	No	No residuum	12	Improved	AIS E, mJOA 18	1

*AIS*, ASIA (American Spinal Injury Association) Impairment Scale; *mJOA*, modified Japanese Orthopedic Association score; *MRI*, magnetic resonance imaging; *CT*, computed tomography

## Discussion

Symptomatic thoracic disc herniations are rare. If symptomatic, a high rate of neurological deficits and progressive myelopathy is common. However, surgery for thoracic disc herniations only makes up for 1–4% of all disc herniation surgeries and is technically challenging and thus associated with a relevant morbidity [3, 4, 6–8, 12]. The anatomy of the thoracic spine hinders an easy access to the anterior-lateral spinal canal, and retraction of the thoracic spinal cord has deleterious consequences [4, 15, 24]. Therefore, access through posterior approaches to thoracic disc herniations remains limited [5, 19]. Traditional anterior approaches like open thoracotomy allow good access to the thoracic anterior-lateral spinal canal [3, 4, 6, 8, 12, 24].

Over the past years, the trend developed towards more minimally invasive surgical approaches using thoracoscopy, which showed a lower complication rate than the before-mentioned more traditional surgical approaches [11, 20, 21]. However, they go with a relevant learning curve of the surgeon. Retropleural approaches to the thoracic spine allow for the shortest way to access the thoracic spinal canal from anterior-laterally, without the necessity for opening the pleural cavity [1, 16]. Combined with endoscopy, they can provide a good access to the spinal canal without the necessity of permanent lung deflation and with a reduced surgery-related morbidity [22, 24]. In a case series of seven patients, a retropleural X-LIF retractor-assisted approach showed feasible without the utilization of neuronavigation or endoscopy, with only minor morbidity [13].

Likewise, the minimally invasive tubular retractor–assisted retropleural approach described in this report allows for a microsurgical retropleural exploration and resection of thoracic disc herniations in a two-center series of six patients. With regard to the most common symptoms of thoracic disc herniations leading to surgical treatment, back and radicular pain did improve and there occurred no neurological deterioration. As surgical complications, a temporary pneumothorax necessitating a postoperative chest tube, and a dural injury necessitating a temporary lumbar drain occurred. Whatsoever, the minimally invasive retropleural exploration with this approach might lower the risk for pleural injury when compared to open approaches.

To ameliorate the safety and feasibility of the described tubular retractor–assisted retropleural approach, the combination with advanced intraoperative state-of-the-art imaging like intraoperative CT, Cone Beam CT (CBCT) or robotic CBCT combined with neuronavigation could prove beneficial in the future [10, 14, 18]. Thus, a safer identification of the anatomical structures could be possible, and the safety of the retropleural preparation might be ameliorated through real-time-control of the right surgical canal. Moreover, the intraoperative assessment of a relevant residual calcified disc herniation following resection is possible with intraoperative CT or CBCT imaging.

The retropleural approach described in this series showed feasible even without the usage of neuronavigation and intraoperative CT imaging. Thus, the approach is not only limited to centres with available intraoperative navigation or advanced intraoperative imaging systems and can be performed broadly with the usage of fluorescence imaging. In summary, a good microsurgical exposure of the disc and neural structures can be achieved minimally invasively from an anterior-lateral direction without the need for an open thoracotomy and permanent lung deflation.

## Conclusion

Using the tubular retractor–assisted retropleural approach, a good microsurgical exposure of the disc and neural structures can be achieved from an anterior-lateral direction without the need for open thoracotomy and permanent lung deflation. In the six treated patients in this case series, control-imaging showed the disc herniation to be removed near complete in all cases without relevant residuum and without residual compression of the neural structures.

## Data Availability

Supporting data is available from the corresponding author upon reasonable request.

## Abbreviations

*AIS*: ASIA Impairment Scale
*ASIA*: American Spinal Injury Association
*BMI*: Body mass index
*CBCT*: Cone beam computed tomography
*CT*: Computed tomography
*Hb*: Hemoglobin
*Hct*: Hematocrit
*ICU*: Intensive care unit
*mJOA*: Modified Japanese Orthopedic Association score
*MRI*: Magnetic resonance imaging
*Th*: Thoracic vertebra
*X-LIF*: Extreme lateral interbody fusion
